# Draft genome sequences of two *Proteus mirabilis* isolates recovered from a municipal wastewater treatment plant in Ontario, Canada

**DOI:** 10.1128/MRA.00559-23

**Published:** 2023-09-29

**Authors:** William Botschner, Hailey Davidson, Opeyemi U. Lawal, Valeria R. Parreira, Lawrence Goodridge

**Affiliations:** 1 Canadian Research Institute for Food Safety, University of Guelph, Guelph, Ontario, Canada; 2 Guelph Wastewater Epidemiology Laboratory for Public Health, University of Guelph, Guelph, Ontario, Canada; Loyola University Chicago, Chicago, Illinois, USA

**Keywords:** *Proteus mirabilis*, wastewater, bacteriophages

## Abstract

*Proteus mirabilis* is a Gram-negative bacterium that is frequently implicated in urinary tract infections in humans and companion animals and has also been associated with foodborne infections in several countries. Here, we report the draft genome sequences of two *P. mirabilis* isolates recovered from municipal wastewater.

## ANNOUNCEMENT

*Proteus mirabilis* is a Gram-negative motile bacterium that is part of the gastrointestinal flora of humans and animals, including chicken and pigs. It is frequently implicated in urinary tract infections and bacteremia (1, 2), and has been associated with foodborne infections in several countries (3–5). The presence of *P. mirabilis* in retail meat products could suggest an emerging food safety risk (1, 2). Here, we present the draft genomes of two *P. mirabilis* (V1 and V4) isolates recovered from a wastewater sample as part of a larger program that employs wastewater-based epidemiology to track foodborne pathogens.

Wastewater sample (500 mL) was collected from a municipal wastewater treatment plant in Guelph, Ontario (43.52276725 N 80.26613714 W), Canada. Sample was pumped (average flow rate: 60 mL/min) through a disposable K-Cup paper filter that was housed in a 3D-printed cartridge. The retentate was enriched with 5 mL tryptic soy broth and plated onto thiosulfate-citrate-bile salts-sucrose agar and incubated at 37°C overnight. Several distinct isolated colonies with typical *P. mirabilis* morphology (6) were subcultured on tryptic-soy agar and were incubated for 24 h at 37°C. DNA was extracted from a pure colony using the DNEasy Blood and Tissue Kit (Qiagen). DNA libraries were prepared using the Illumina DNA prep tagmentation and IDT for Illumina DNA/RNA UD indexes. Paired-end (2 × 150 bp) sequencing was performed on the Illumina MiniSeq system. Sequencing of isolates V1 and V4 yielded 707,623 and 1,622,998 paired-end reads, respectively. Raw reads were quality filtered, trimmed, and assembled as previously described (7, 8). Assembly quality was assessed using QUAST v5.2 (9). Genome annotation was performed using the NCBI Prokaryotic Genome Annotation Pipeline v6.3 (10). The resistome was determined using AMRFinder plus v3.10.45 (11), while prophages and plasmids were identified using PHASTER (12) and MOB-suite v3.1 (13), respectively. All bioinformatics tools were used with default parameters. Using *k-mer*-based species taxonomic classification with the Kraken2 database (14), isolates V1 and V4 were identified as *P. mirabilis*. The draft genomes of *P. mirabilis* isolates V1 and V4 on average yielded 45 contigs, 38.62% GC content, and 3,943,985 bp genome size, with at least 48× coverage (Table 1). The two *P. mirabilis* isolates were highly similar with average nucleotide identity of 99.8% as determined by ANItools (14). Isolate V1 contained 3,551 protein-coding sequences and 72 RNAs, while V4 had 3,544 protein-coding sequences and 82 RNAs. Isolates V1 and V4 carried two genes coding for resistance to tetracycline (*tetJ*) and chloramphenicol (*catA4*). While no plasmids were detected in the draft genomes, three intact temperate phages (phage 1: 20.8 kb, phage 2: 38.1 kb, and phage 3: 43.3 kb) were identified (Table 1). The draft genomes of *P. mirabilis* presented here will contribute to the growing genomic data sets available for comparative genomic analysis and evolutionary studies of this bacterium.

**TABLE 1:** Summary of sequence metrics of the two *P. mirabilis* isolates recovered from municipal wastewater

Isolate ID	Reads	Contigs	Genome size (bp)	N50 (bp)	Coverage	Resistome	Phages (intact)	SRA accession	GenBank accession
*P. mirabilis* V1	707,623	49	3,940,476	202,420	48x	*tetJ, catA4*	3 (phage1-contig 1: 215,529–236,417; phage 2-contig 9: 72,427–110,607; phage3-contig 1: 154,257–197,576)	SRR24313739	JARYZU000000000
*P. mirabilis* V4	1,622,998	41	3,947,494	231,972	114×	*tetJ, catA4*	3 (phage1-contig 3: 216,066–236,954; phage 2-contig 11: 72,627–110,807; phage 3-contig 3: 154,920–198,113)	SRR24313738	JARYZV000000000

## Data Availability

This Whole-Genome Shotgun project has been deposited at DDBJ/ENA/GenBank under the accession JARYZU000000000 and JARYZV000000000 and SRA accession SRR24313739 and SRR24313738 for *P. mirabilis* V1 and V4 strains, respectively. The versions described in this paper are JARYZU000000000 and JARYZV000000000.

## References

[1] Marques C, Belas A, Menezes J, Moreira da Silva J, Cavaco-Silva P, Trigueiro G, Gama LT, Pomba C. 2021. Human and companion animal Proteus Mirabilis sharing. Microbiol Res 13:38–48. doi:10.3390/microbiolres13010003

[2] Marques C, Belas A, Franco A, Aboim C, Gama LT, Pomba C. 2018. Increase in antimicrobial resistance and emergence of major international high-risk clonal lineages in dogs and cats with urinary tract infection: 16 year retrospective study. J Antimicrob Chemother 73:377–384. doi:10.1093/jac/dkx401 29136156 PMC5890753

[3] Ma WQ, Han YY, Zhou L, Peng WQ, Mao LY, Yang X, Wang Q, Zhang TJ, Wang HN, Lei CW. 2022. Contamination of Proteus mirabilis harbouring various clinically important antimicrobial resistance genes in retail meat and aquatic products from food markets in China. Front Microbiol 13:1086800. doi:10.3389/fmicb.2022.1086800 36590410 PMC9802577

[4] Wang Y, Zhang S, Yu J, Zhang H, Yuan Z, Sun Y, Zhang L, Zhu Y, Song H. 2010. An outbreak of Proteus mirabilis food poisoning associated with eating stewed pork balls in brown sauce, Beijing. Food Control 21:302–305. doi:10.1016/j.foodcont.2009.06.009

[5] Gong Z, Shi X, Bai F, He X, Zhang H, Li Y, Wan Y, Lin Y, Qiu Y, Chen Q, Hu Q, Cao H. 2019. Characterization of a novel diarrheagenic strain of Proteus mirabilis associated with food poisoning in China. Front Microbiol 10:2810. doi:10.3389/fmicb.2019.02810 31921012 PMC6921692

[6] Nakahara A, Shimada Y, Wakita J, Matsushita M, Matsuyama T. 1996. Morphology diversity of the colony produced by Proteus mirabilis. J Phys Soc jpn 65:2700–2706. doi:10.1143/JPSJ.65.2700

[7] Bryan N, Anderson R, Lawal OU, Parreira VR, Goodridge L. 2023. Draft genome sequence of Exiguobacterium sp strain N5, isolated from a recreational freshwater kettle Lake in Ontario. Microbiol Resour Announc 12:e0126122. doi:10.1128/mra.01261-22 36880761 PMC10112067

[8] Bryan N, Anderson R, Lawal OU, Parreira VR, Goodridge L, Maresca JA. 2023. Draft genome sequence of Bacillus anthracis N1, isolated from a recreational freshwater kettle lake in Ontario. Microbiol Resour Announc 12:e0126222. doi:10.1128/mra.01262-22 36912633 PMC10112256

[9] Gurevich A, Saveliev V, Vyahhi N, Tesler G. 2013. QUAST: quality assessment tool for genome assemblies. Bioinformatics 29:1072–1075. doi:10.1093/bioinformatics/btt086 23422339 PMC3624806

[10] Tatusova T, DiCuccio M, Badretdin A, Chetvernin V, Nawrocki EP, Zaslavsky L, Lomsadze A, Pruitt KD, Borodovsky M, Ostell J. 2016. NCBI prokaryotic genome annotation pipeline. Nucleic Acids Res 44:6614–6624. doi:10.1093/nar/gkw569 27342282 PMC5001611

[11] Feldgarden M, Brover V, Haft DH, Prasad AB, Slotta DJ, Tolstoy I, Tyson GH, Zhao S, Hsu CH, McDermott PF, Tadesse DA, Morales C, Simmons M, Tillman G, Wasilenko J, Folster JP, Klimke W. 2019. Validating the amrfinder tool and resistance gene database by using antimicrobial resistance genotype-phenotype correlations in a collection of isolates. Antimicrob Agents Chemother 63:e00483-19. doi:10.1128/AAC.00483-19 31427293 PMC6811410

[12] Arndt D, Grant JR, Marcu A, Sajed T, Pon A, Liang Y, Wishart DS. 2016. PHASTER: a better, faster version of the PHAST phage search tool. Nucleic Acids Res 44:W16–W21. doi:10.1093/nar/gkw387 27141966 PMC4987931

[13] Robertson J, Nash JHE. 2018. MOB-suite: software tools for clustering, reconstruction and typing of plasmids from draft assemblies. Microb Genom 4:e000206. doi:10.1099/mgen.0.000206 30052170 PMC6159552

[14] Han N, Qiang Y, Zhang W. 2016. Anitools web: a web tool for fast genome comparison within multiple bacterial strains. Database (Oxford) 2016:baw084. doi:10.1093/database/baw084 27270714 PMC4911789

